# Isolated absence of nasal bone in 1 fetus in a dizygotic pregnancy after in vitro fertilization: A case report

**DOI:** 10.1097/MD.0000000000022558

**Published:** 2020-10-02

**Authors:** Xun Zeng, Xiaohong Li, Lang Qin, Wei Huang, Song Jin, Haiyan Yu

**Affiliations:** a Department of Obstetrics and Gynecology, West China Second University Hospital, Sichuan University; b Key Laboratory of Birth Defects and Related Diseases of Women and Children, Sichuan University, Ministry of Education, Chengdu, China.

**Keywords:** in vitro fertilization, dizygotic pregnancy, fetal nasal bone, ultrasound, prenatal diagnosis

## Abstract

**Rationale:**

During ultrasound prenatal screening, absence of the fetal nasal bone is used as a marker for common aneuploidies in singleton pregnancies. However, its application in multiple pregnancies is less sensitive and more challenging owing to difficulties in obtaining adequate views of the fetal face.

**Patient concerns:**

A 38-year-old woman with dichorionic-diamniotic (DCDA) pregnancy and a history of in vitro fertilization and embryo transfer was referred to our hospital with the absence of the nasal bone noted on ultrasound images obtained during the second trimester in 1 fetus.

**Diagnosis:**

Prenatal sonographic examination revealed the absence of the nasal bone in 1 fetus in the DCDA gestation. Amniocentesis performed on the dual amniotic sacs revealed normal karyotypes for each twin. The absence of the nasal bone was confirmed on a radiograph obtained postnatally in 1 infant.

**Interventions:**

The mother underwent routine outpatient care according to the gestational age and successfully delivered following lower-segment cesarean section.

**Outcomes:**

Two live infants were uneventfully delivered. Radiography confirmed the absence of the nasal bone in 1 of the newborns on postnatal day 3. The infants were followed up until 2 years and 9 months of age, which revealed normal appearance and eating and breathing functions.

**Lessons:**

Prenatal diagnosis of the absence of nasal bone in 1 fetus of DCDA pregnancy has rarely been reported. Although a fetus with the absence of the nasal bone in DCDA gestation poses a significant risk of aneuploidy, it is acceptable when the defect is an isolated anomaly after ruling out genetic abnormalities. Appropriate consultation should be provided for these patients.

## 1 Introduction

In the last 3 decades, owing to the use of assisted reproductive techniques and delayed childbearing, the numbers of pregnancies in women at advanced maternal age and incidence rates of multiple pregnancies are increasing, especially for dizygotic twins. Twin pregnancies have higher risks of pregnancy-related complications and chromosomal abnormalities than singleton gestations.[^1^,^2^] Meanwhile, advanced maternal age increases the risk of chromosomal aneuploidy.^[3]^

Prenatal screening for chromosomal abnormalities has been widely used in many obstetric units; however, that in a twin pregnancy is complicated. Fetal nasal bone (FNB), as a frequent demand of prenatal ultrasound screening, is a genetic sonographic marker detected in the first or second trimester during prenatal ultrasonography in singleton pregnancy. The measurement of FNB enables fetus-specific identification of those at a high risk of aneuploidy. Failed visualization of the FNB in singleton pregnancies is a common facial feature of fetuses with trisomy 21 and other aneuploidies, such as trisomy 13 and 18.[^4^,^5^] Prenatal diagnosis of the absence of the nasal bone in 1 fetus of a dichorionic-diamniotic (DCDA) pregnancy with normal karyotype is rarely reported.

In this report, we present a case of a 38-year-old woman with DCDA pregnancy via in vitro fertilization (IVF) and embryo transfer who revealed absence of the nasal bone in 1 fetus on ultrasonography obtained during the second trimester. Although amniocentesis showed normal karyotypes for each twin, postnatal radiograph confirmed the absence of the nasal bone in 1 infant. On subsequent follow-up assessments, the infant showed normal appearance and normal eating and breathing functions.

## 2 Case presentation

**Figure 1:**
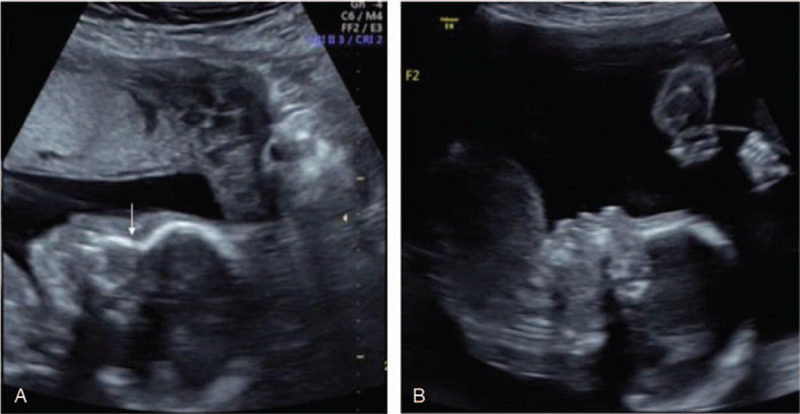
Ultrasound image showing a dichorionic-diamniotic twin pregnancy discordant for the absence of the fetal nasal bone (FNB) at 21 + 2 weeks’ gestation. Sagittal view of the fetal face demonstrating normal nasal bone in twin A (arrow) (A) and an abnormal profile with the absence of the nasal bone in the affected twin B (B).

**Figure 2:**
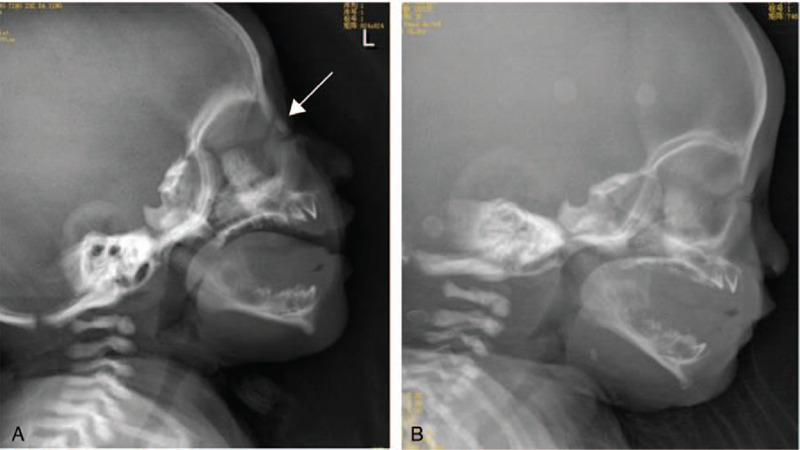
Radiograph shows normal nasal bone in the newborn twin A (arrow) (A) and absence of the nasal bone in newborn twin B (B) on postnatal day 3.

A 38-year-old nulliparous woman, gravida 4, para 3, with a previous induced abortion in the first trimester and 2 ectopic pregnancies was referred at 21 + 2 weeks of gestation with absence of the nasal bone in 1 fetus. The couple reported infertility problems, and the woman has conceived via IVF with transfer of 3 cleavage embryos. Her medical history was negative for hypertension, diabetes mellitus, and other conditions. She had undergone bilateral salpingectomy due to ectopic pregnancies. She denied history of any congenital anomalies or chronic conditions. The couple was healthy and had no history of exposure to any medications or other teratogens. She had no remarkable family history. Ultrasound taken during the first trimester showed 2 different gestational sacs, indicating dichorionic twins. The IVF-derived gestational ages matched those determined by ultrasound measurements in the first trimester. The twins showed normal nuchal translucency during screening in the first trimester. At 19 + 1 weeks of gestation, amniocentesis was conducted on the dual amniotic sacs because of advanced age, which revealed normal karyotypes for each twin. Prenatal sonography showed absence of the nasal bone (Fig. 1) in twin B at 21 + 2-weeks of gestation and no other structural anomalies. Subsequently, extensive prenatal genetic counseling and discussions with the laryngologist about potential neonatal clinical issues were conducted. In light of this uncertainty, the couple made the difficult decision to continue the pregnancy. The couple refused to undergo magnetic resonance imaging (MRI), and a serial follow-up scan was selected as the alternative management strategy. Sonography at 25 + 4 weeks confirmed the absence of the nasal bone in twin B. An ultrasound performed 5 weeks later also did not show the nasal bone in twin B. The mother subsequently received routine outpatient care according to the gestational age. Subsequent reports of ultrasonography were reviewed for anomalies, and no abnormalities were seen in growth, amniotic fluid volume, and other ultrasonic indexes. The pregnant woman delivered 2 live babies weighing 2030 g and 1940 g by cesarean section at 36 + 1 weeks of gestation. Twin B showed normal external nose and had no difficulties in breastfeeding and breathing after birth. Subsequently, the absence of the nasal bone was confirmed on a radiograph in twin B on postnatal day 3 (Fig. 2). At the time of submission of this manuscript, the twin B was 2 years and 9 months old and showed normal breathing and eating functions.

## 3 Discussion

The nasal bones are a pair of small oblong bones that form the bridge of the nose, which can be detected first in the 10th week of gestation and is clearly visible in the second trimester when it becomes apparent histologically.^[6]^ The absence of the FNB is caused by failure of development of the centers of ossification. Inability to identify them below the skin of the nasal bridge in a magnified mid-sagittal view of the head is considered to be a significant ultrasound marker for chromosomal anomalies in singleton pregnancies.^[7]^ A close association has been reported between the absence of the nasal bone and aneuploidy, including trisomy 13,18, and 21, in singleton pregnancies at the first- or second-trimester screening, which was demonstrated in radiological and histomorphological studies.[^4^,^8^]

We reviewed the literature on risk factors that might explain the absence of the nasal bone in 1 of the twins. The following iatrogenic factors have been reported: maternal advanced age,^[3]^ multiple gestations, and use of assisted reproductive technology.[^9^,^10^] All these factors were present in our patient. In dizygotic multiple pregnancies, an additional fetus proportionally increases the risk of aneuploidy. Meanwhile, the advanced maternal age also increases the risk. A fetus with the absence of the nasal bone in twin pregnancy poses a significant risk of aneuploidy and structural defects as potential sequelae, if left unrecognized. Therefore, dual amniocentesis was performed when discordant twins were suspected. However, both fetuses showed normal karyotypes. Remarkably, nasal bone measurement is limited in sensitivity and is challenging in twins than that in singleton pregnancies^[11]^ because of difficulties in obtaining the mid-sagittal plane of the fetal face owing to their positions. There is a paucity of studies on nasal bone assessment in multiple pregnancies. Only 1 retrospective study^[12]^ reported that first trimester screening of chromosomal abnormalities using nuchal translucency and nasal bone assessment in 206 twin pregnancies found the absence of the nasal bone in 1 fetus in 4 DCDA pregnancies. The twins had normal chromosomes in only 1 twin gestations, while the other 3 cases showed aneuploidy in the fetuses, including trisomy 18 and 21. However, the study did not report any details on perinatal outcomes and follow-ups.

To the best of our knowledge, there has been no reported case of the absence of the nasal bone in 1 fetus in IVF DCDA pregnancies. In our report, isolated absence of the nasal bone in 1 fetus of the DCDA twins with normal karyotypes was found on prenatal ultrasound screening and confirmed on postnatal radiograph. The newborn with an absence of the nasal bone had normal appearance and showed no difficulties in breathing and eating on subsequent follow-ups until the age of 2 years and 9 months. Long-term follow-up is required when the absence of FNB is present alone. Considering that the infant did not have any congenital deformities in other regions of the body and no family history of similar problems, it could be attributed to localized abnormality in the development of the germ cells.

## 4 Conclusion

Although a fetus with absence of the nasal bone in DCDA pregnancy poses a significant risk of aneuploidy, it is acceptable when the defect is an isolated anomaly after ruling out genetic abnormalities. In this case, the absence of the nasal bone was confirmed on postnatal radiograph; however, the infant showed no feeding or respiratory abnormalities from birth until 2 years and 9 months of age. Appropriate consultation should be provided to such patients during pregnancy.

## Author contributions

**Resources:** Xun Zeng, Haiyan Yu, Song Jin.

**Supervision:** Wei Huang, Haiyan Yu.

**Writing – original draft:** Xun Zeng, Xiaohong Li.

**Writing – review & editing:** Lang Qin.
